# Sildenafil restores endothelial function in the apolipoprotein E knockout mouse

**DOI:** 10.1186/1479-5876-11-3

**Published:** 2013-01-05

**Authors:** Camille M Balarini, Marcos A Leal, Isabele B S Gomes, Thiago M C Pereira, Agata L Gava, Silvana S Meyrelles, Elisardo C Vasquez

**Affiliations:** 1 Dept. of Physiological Sciences, Health Sciences Center, Federal University of Espirito Santo, Vitoria, ES, Brazil; 2 Emescam College of Health Sciences, Vitoria, ES, Brazil; 3 Dept. of Pharmaceutical Sciences, Health Sciences Center, Federal University of Espirito Santo, Vitoria, ES, Brazil; 4 Federal Institute of Education, Science and Technology (IFES), Vila Velha, ES, Brazil; 5 Department of Pharmaceutical Sciences, University of Vila Velha, Vila Velha, ES, Brazil; 6 Biotechnology Graduate Program, Health Sciences Center, Federal University of Espirito Santo, Vitoria, ES, Brazil; 7 Lab Transgenes and Cardiovascular Control, Dept. of Physiological Sciences, Health Sciences Center, Federal University of Espirito Santo, Av Marechal Campos 1468, 29042-755, Vitoria, ES, Brazil

**Keywords:** Atherosclerosis, ApoE knockout mice, Sildenafil, Nitric oxide, Oxidative stress, Endothelial dysfunction, PDE5, cGMP

## Abstract

**Background:**

Atherosclerosis is an inflammatory process of the arterial walls and is initiated by endothelial dysfunction accompanied by an imbalance in the production of reactive oxygen species (ROS) and nitric oxide (NO). Sildenafil, a selective phosphodiesterase-5 (PDE5) inhibitor used for erectile dysfunction, exerts its cardiovascular effects by enhancing the effects of NO. The aim of this study was to investigate the influence of sildenafil on endothelial function and atherosclerosis progression in apolipoprotein E knockout (apoE^−/−^) mice.

**Methods:**

ApoE^−/−^ mice treated with sildenafil (Viagra®, 40 mg/kg/day, for 3 weeks, by oral gavage) were compared to the untreated apoE^−/−^ and the wild-type (WT) mice.

Aortic rings were used to evaluate the relaxation responses to acetylcholine (ACh) in all of the groups. In a separate set of experiments, the roles of NO and ROS in the relaxation response to ACh were evaluated by incubating the aortic rings with L-NAME (NO synthase inhibitor) or apocynin (NADPH oxidase inhibitor). In addition, the atherosclerotic lesions were quantified and superoxide production was assessed.

**Results:**

Sildenafil restored the vasodilator response to acetylcholine (ACh) in the aortic rings of the apoE^−/−^ mice. Treatment with L-NAME abolished the vasodilator responses to ACh in all three groups of mice and revealed an augmented participation of NO in the endothelium-dependent vasodilation in the sildenafil-treated animals. The normalized endothelial function in sildenafil-treated apoE^−/−^ mice was unaffected by apocynin highlighting the low levels of ROS production in these animals. Moreover, morphological analysis showed that sildenafil treatment caused approximately a 40% decrease in plaque deposition in the aorta.

**Conclusion:**

This is the first study demonstrating the beneficial effects of chronic treatment with sildenafil on endothelial dysfunction and atherosclerosis in a model of spontaneous hypercholesterolemia. These data indicate that the main mechanism of the beneficial effect of sildenafil on the endothelial function appears to involve an enhancement of the NO pathway along with a reduction in oxidative stress.

## Introduction

Atherosclerosis, which can be defined as a chronic and progressive disease characterized by an inflammatory response of the arterial wall, is still a leading cause of death, mainly in the Western world [1,2]. The development of atherosclerotic plaques is influenced by oxidative stress, an imbalance between the accumulation of reactive oxygen species (ROS) mainly produced by NADPH oxidase and limited antioxidant defenses; this imbalance compromises nitric oxide (NO) availability and leads to endothelial dysfunction [3,4]. Early studies suggest that the correction of endothelial dysfunction results in a better prognosis [5], supporting the need for drugs to improve endothelial function and to reduce the progression of atherosclerosis [6].

Sildenafil, which has largely been used for erectile dysfunction and pulmonary hypertension [7], is being used to enhance *in vivo* NO signaling in other vascular diseases as well [6]. This drug increases the bioavailability of the intracellular second messenger of NO (cGMP) by inhibition of phosphodiesterase-5 (PDE5). Sildenafil was recently shown to improve endothelial function in coronary arterial disease as well as in diabetes and to reduce oxidative stress in many tissues [8-11]. However, the drug has not yet been tested in experimental models of atherosclerosis.

The apolipoprotein E knockout (apoE^−/−^) mouse developed two decades ago [12,13], exhibits spontaneous hypercholesterolemia, accompanied by endothelial dysfunction similar to that observed in humans [4,14,15]. Therefore, the apoE^−/−^ mouse seems to be a suitable genetic model to test new therapeutic approaches for the treatment of vascular diseases observed in dyslipidemias [16].

The aim of the present study was to test the hypothesis that chronic sildenafil treatment could revert or reduce the endothelial dysfunction and the progression of atherosclerosis observed in large vessels of apoE^−/−^ mice. Our data show that this drug was able to restore the endothelial function in the aorta of apoE^−/−^ mice, primarily by enhancing the activation of the NO pathway and by decreasing ROS production.

## Materials and methods

### Animals

Experiments were conducted in adult (18-week-old) male apoE^−/−^ mice, which have a C57BL/6J genetic background, and on C57BL/6J wild-type (WT) mice, bred and maintained in the animal care facility at the Laboratory of Transgenes in the Health Sciences Center at the Federal University of Espirito Santo, Brazil. Mice were housed in individual plastic cages with a controlled temperature (22-23°C) and humidity (60%) and were exposed to a 12:12-h light–dark cycle. All experimental procedures were performed in accordance with the guidelines for the care and handling of laboratory animals as recommended by the National Institutes of Health (NIH), and study protocols were previously approved by the Institutional Animal Care Committee (CEUA-Emescam, Protocol # 007/2010).

To accelerate and aggravate the spontaneous hyperlipidemia and progression of endothelial dysfunction and atherosclerosis in apoE^−/−^ mice, the animals were fed a Western-type diet for 10 weeks, starting at 8 weeks of age (AIN93G modified diet, Rhoster, Brazil). After 7 weeks (15-week-old), the mice were divided into three different groups: (a) apoE^−/−^ mice treated with the PDE5 inhibitor sildenafil (Viagra®, 40 mg/kg/day, for 3 weeks, by oral gavage), (b) apoE^−/−^ mice treated with the vehicle and (c) WT control mice, treated with the vehicle.

### Measurement of plasma lipids

Blood samples were obtained by puncturing the right ventricle of the animals euthanized with an overdose of thiopental sodium anesthetic. The blood was immediately transferred to a tube containing heparin and centrifuged at 3000 RPM for 10 min. Plasma was separated immediately and kept at −20°C until assayed. Total plasma cholesterol, high density lipoproteins (HDL), low density lipoproteins (LDL) and triglycerides were determined using commercial colorimetric assay kits (Bioclin, Belo Horizonte, Brazil). Very low density lipoproteins (VLDL) and intermediate density lipoproteins (IDL) were estimated by subtracting HDL and LDL from total serum cholesterol.

### Vascular function

After the treatment, animals were anesthetized with a sodium thiopental injection (200 mg/kg, i.p.), blood samples were collected and the thoracic aorta was removed and immediately placed in modified Krebs buffer of the following composition (in mM): NaCl 115, KCl 4.7, CaCl_2_·2H_2_O 2.5, MgSO_4_·7H_2_O 1.2, KH_2_PO_4_ 1.2, NaHCO_3_ 25, EDTA 0.01 and glucose 11.1. Loose connective tissue in the adventitia was carefully removed to avoid damage, and the vessel was cut into rings (3 to 4 mm thick). The aortic rings were then mounted on stainless steel triangles, suspended in vertical organ baths containing 5 ml Krebs solution, maintained at a resting tension of 0.5 g at 37°C, pH 7.4 and continuously gassed with a mixture of 95% O_2_ and 5% CO_2_. The rings were connected to force transducers to measure isometric tension through a computerized acquisition system (MP100, Biopac Systems Inc., Santa Barbara, USA).

After a 60-min equilibration period, aortic rings were exposed to KCl (125 mM) to assess their maximal tension. The endothelial function was then verified by acetylcholine (ACh)-induced relaxation (10 μM) after pre-contraction with phenylephrine (PE, 10 μM); the endothelium was considered functional when the relaxation was greater than 50%. After a washout period of 30 minutes, endothelium-dependent relaxation responses were tested by concentration-response curves to cumulative concentrations of ACh (100 pM – 30 μM).

In a separate set of experiments, the role of NO and ROS in the relaxation response to ACh were evaluated by incubation of the aortic rings with the non-specific NO synthase (NOS) inhibitor N(G)-nitro-L-arginine methyl ester (L-NAME, 100 μM), or the NADPH oxidase inhibitor apocynin (300 μM) that were added to the vessel bath 20 minutes prior to assessing the concentration-response curves. The area under the curve (AUC) for each of the responses of the aortic rings to ACh in the presence or absence of L-NAME was calculated, and these results were expressed in arbitrary units.

### Quantification of the atherosclerotic lesion

In another set of animals, the mice were euthanized with a sodium thiopental overdose (200 mg/kg, i.p.), and the left ventricle was perfused with 0.1 M phosphate-buffered saline (PBS, pH 7.4), followed by a 4% formaldehyde solution in PBS at a pressure of 100 mm Hg. The aortic root and a portion of the ascendant aorta were removed, placed in PBS, dissected free from fat and adhering perivascular tissue, embedded in OCT compound and cross-sectioned on a cryostat (Jung CM1800; Leica, Wetzlar, Germany) at a thickness of 7 μm. For each animal, aorta cross-sections were mounted on gelatin-coated slides and stained with Oil-Red-O (Sigma-Aldrich, St. Louis, MO, USA). Atherosclerotic lesions were quantified using a microscope (Olympus AX70, Olympus, Center Valley, PA, USA) interfaced with a video camera (VKC150, Hitachi, Tokyo, Japan) and an image analysis system (Image J, Public Domain). The lesion area per mouse was calculated by an investigator who was blinded to the experimental protocol and was expressed as a percentage of the lumen occupied by the atherosclerotic plaque.

### Detection of superoxide production

Unfixed frozen sections from the aorta were cut into 7-μm-thick sections and mounted on gelatin coated glass slides. Samples were incubated with the oxidative fluorescent dye dihydroethidium (DHE, 2 μmol/L) in a modified Krebs's solution (containing 20 mM HEPES), in a light-protected humidified chamber at 37°C for 30 min, to detect superoxide. The intensity of fluorescence was quantified in the tissue sections using a microscope (Nikon Eclipse TI, Nikon Instruments Inc., Melville, NY, USA) by an investigator blinded to the experimental protocol.

### Statistical analysis

Values were expressed as the means ± S.E.M. Relaxation responses to ACh and SNP were expressed as the percentage of dilation relative to the maximal pre-contraction level. For each concentration-response curve, the maximum effect (R_max_) and the log of the concentration of agonist that produced half of R_max_ (log EC_50_) were calculated using nonlinear regression analysis. The sensitivities of the agonists were expressed as pEC_50_ (−log EC_50_). Statistical comparisons between the different groups were performed by one-way or two-way analysis of variance (ANOVA) followed by Bonferroni’s *post hoc* test. The statistical analyses were performed using Prism software (Prism 5, GraphPad Software, Inc., San Diego, CA, USA). A value of p < 0.05 was regarded as statistically significant.

## Results

Table 1 summarizes the results of plasma lipid profile of the three groups of animals. As expected, the apoE^−/−^ vehicle group which received a Western type diet for 10 weeks, showed a significant increase in triglycerides (4-fold) along with a marked increase in total plasma cholesterol (14-fold) and VLDL + IDL (42-fold), when compared to the WT mice. On the other hand, HDL was significantly decreased (4-fold) in the apoE^−/−^ mice compared to the WT mice. Sildenafil treatment did not change the profile in apoE^−/−^ animals.

**Table 1:** Lipid profiles of vehicle- and sildenafil-treated apolipoprotein E-deficient (apoE^−/−^) mice fed a Western-type diet and wild-type control (WT)

**Parameter**	**WT**	** apoE**^**−/−**^	
		**Vehicle**	**Sildenafil**
Triglycerides (mg/dL)	61 ± 9	241 ± 36**	196 ± 46*
Total plasma cholesterol (mg/dL)	94 ± 5	1308 ± 145**	1310 ± 223**
LDL (mg/dL)	23 ± 6	180 ± 29**	190 ± 48**
HDL (mg/dL)	58 ± 4	15 ± 4**	10 ± 1**
VLDL + IDL (mg/dL)	26 ± 5	1115 ± 28**	1111 ± 48**

Values are mean ± SEM of 5–10 mice per group. *p < 0.05 and **p < 0.01 *vs.* WT (one-way ANOVA).

**Figure 1:**
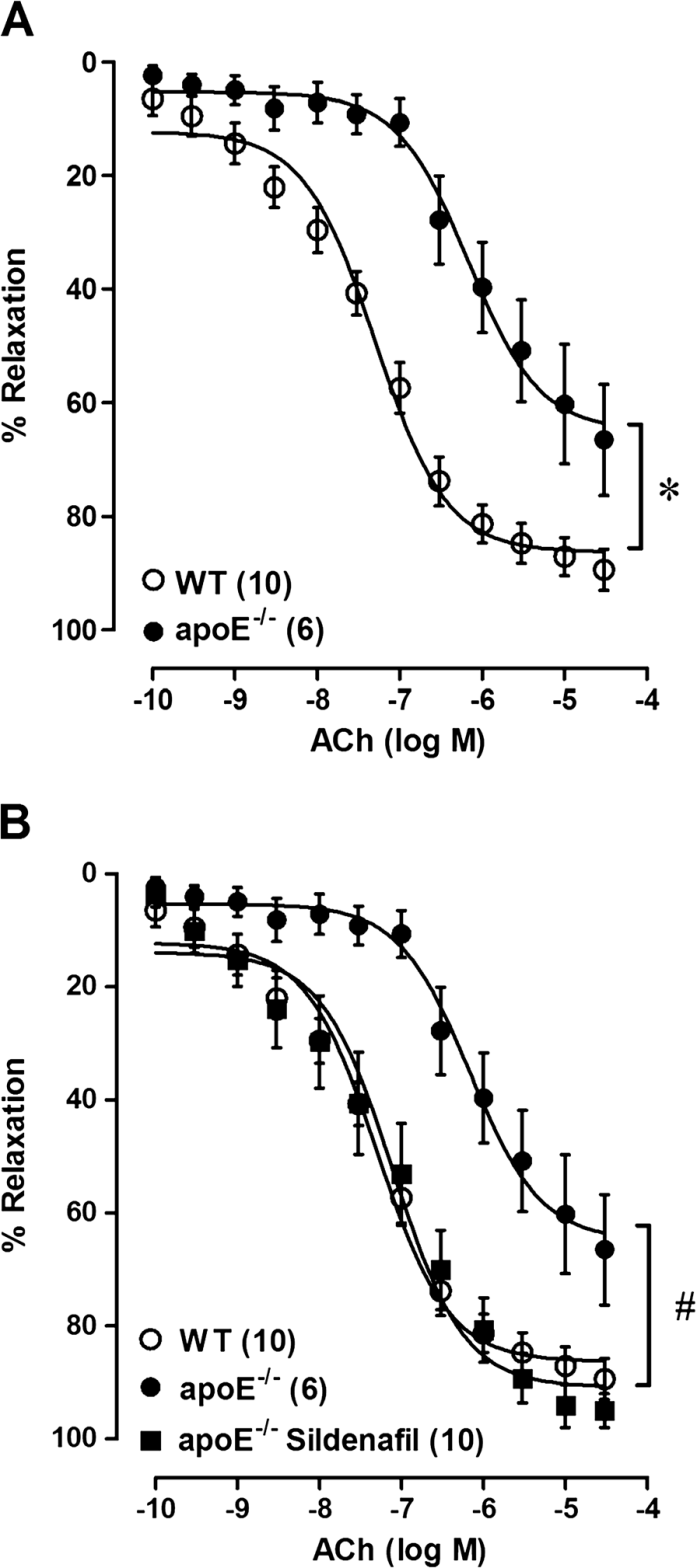
**Effect of sildenafil treatment on the endothelial function of apoE^−/−^ mice. **(**A**) Acetylcholine (ACh)-induced endothelium-dependent relaxation in aortic rings, comparing untreated apoE^−/−^ mice with wild-type (WT) control mice. (**B**) Effect of the treatment with sildenafil (40 mg/kg/day for 3 weeks) on ACh-induced vasodilation in apoE^−/−^ mice compared to untreated apoE^−/−^ and to WT mice. Values are the means ± SEM. *p < 0.05 vs. WT; ^#^p < 0.05 vs. untreated apoE^−/−^ (two-way ANOVA).

The maximal vascular response (R_max_) and sensitivity (pEC_50_) to ACh were significantly decreased in the aortas of untreated apoE^−/−^ animals, compared to those of the WT mice (Table 2, Figure 1A). This impaired ACh-induced relaxation response in the apoE^−/−^ mice was completely restored on sildenafil treatment to values similar to those observed in the WT mice (Table 2, Figure 1B).

**Table 2:** Effect of L-NAME and apocynin on the efficacy of and sensitivity to acetylcholine in isolated aortic rings from wild-type control (WT) mice and from vehicle- and sildenafil-treated apolipoprotein E-deficient (apoE^−/−^) mice

**Curve parameter**	**WT**	** apoE**^**−/−**^	
		**Vehicle**	**Sildenafil**
***Acetylcholine***			
R_max_ (%)	87 ± 3.6	66 ± 9.7*	95 ± 3.1^#^
pEC_50_	7.3 ± 0.1	6.1 ± 0.1**	7.2 ± 0.3^##^
***Acetylcholine + L-NAME***			
R_max_ (%)	11 ± 2.0^§§^	18 ± 4.4^§§^	12 ± 2.8^§§^
pEC_50_	8.7 ± 0.5^§^	8.5 ± 0.2^§§^	7.3 ± 1.1
***Acetylcholine + apocynin***			
R_max_ (%)	84 ± 5.2	101 ± 4.6^§§^	94 ± 3.7
pEC_50_	7.3 ± 0.2	7.3 ± 0.2^§§^	7.2 ± 0.2

R_max_, maximal response (efficacy). pEC_50_, the negative logarithm of the concentration required to produce 50% of the R_max_ (sensitivity). Values are mean ± SEM of 5–10 mice per group. *p < 0.05 and **p < 0.01 *vs.* WT and ^#^p < 0.05 and ^##^p < 0.01 *vs.* apoE^−/−^ vehicle (one-way ANOVA); ^§^p < 0.05 and ^§§^p < 0.01 *vs.* respective control without blockade (two-way ANOVA).

**Figure 2:**
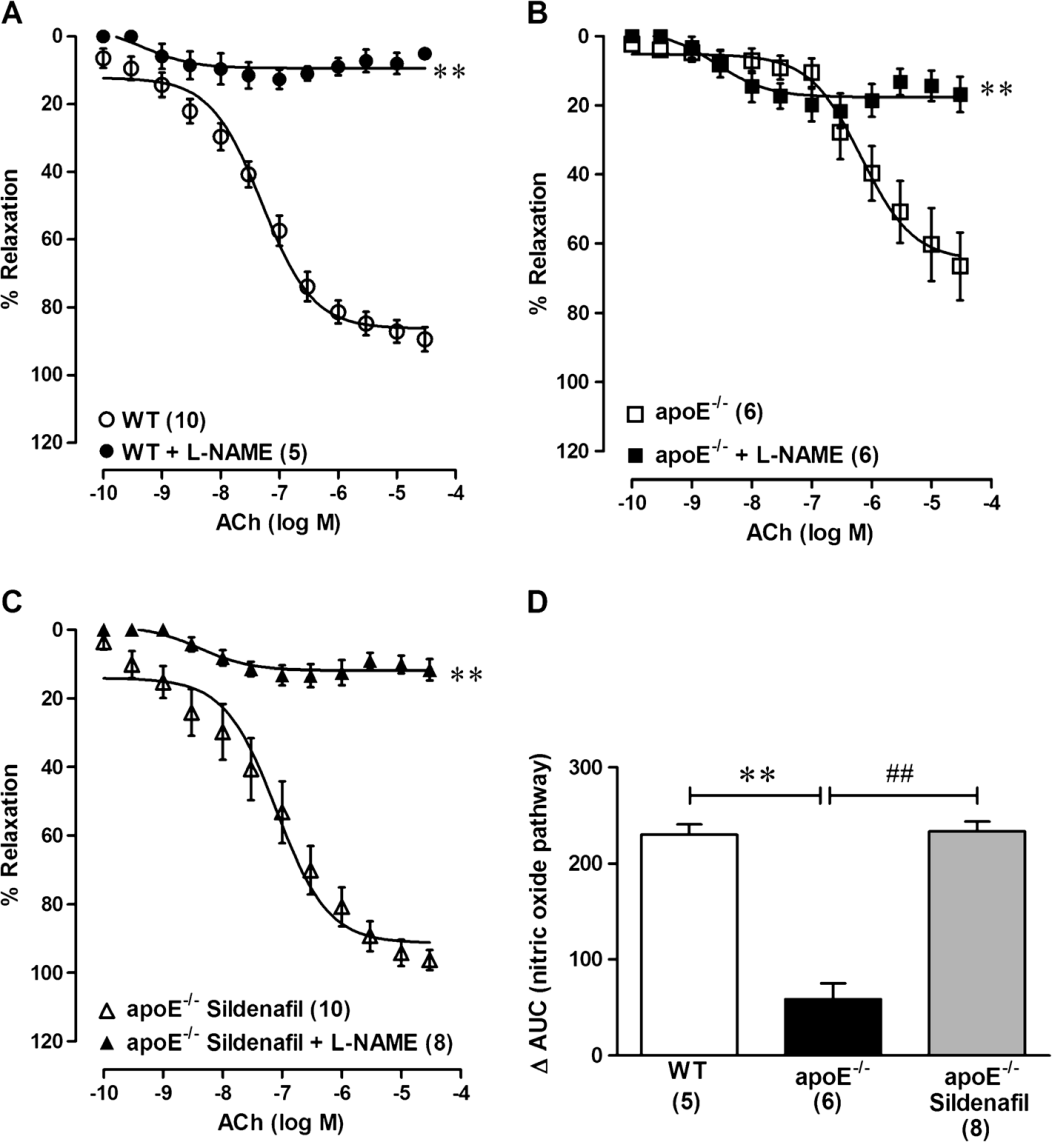
**Participation of nitric oxide in ACh-induced vasodilation.** Concentration-response curves show acetylcholine (ACh)-induced endothelium-dependent relaxation in aortic rings under the blockade of nitric oxide synthase with L-NAME in wild-type (WT) mice (**A**), untreated apoE^−/−^ mice (**B**) and sildenafil-treated apoE^−/−^ mice (**C**). Bar graph (**D**) shows the difference in the area under the curve (AUC) of the response of aortic rings to ACh with and without L-NAME. A decreased difference in the AUC indicates an impaired response to ACh. Values are the means ± SEM. **p < 0.01 vs. without L-NAME (**A**, **B** and **C**: two-way ANOVA); **p < 0.01 vs. WT and ^##^p < 0.01 vs. untreated apoE^−/−^ (**D**: one-way ANOVA).

To evaluate the participation of NO in endothelium-dependent vasodilator responses, aortic rings were incubated with L-NAME prior to ACh concentration-response curves. In all groups, L-NAME did block the vasodilator response elicited by ACh (Table 2, Figures 2A, 2B, 2C). Moreover, analysis of the difference of the AUC revealed that the participation of NO in the relaxation response to ACh was significantly reduced in the untreated apoE^−/−^ mice when compared to the WT mice (58 ± 17 vs. 230 ± 11, p < 0.01). Sildenafil treatment recovered the normal ACh-induced relaxation in apoE^−/−^ mice when compared to untreated apoE^−/−^ mice (233 ± 10, p < 0.01, Figure 2D).

**Figure 3:**
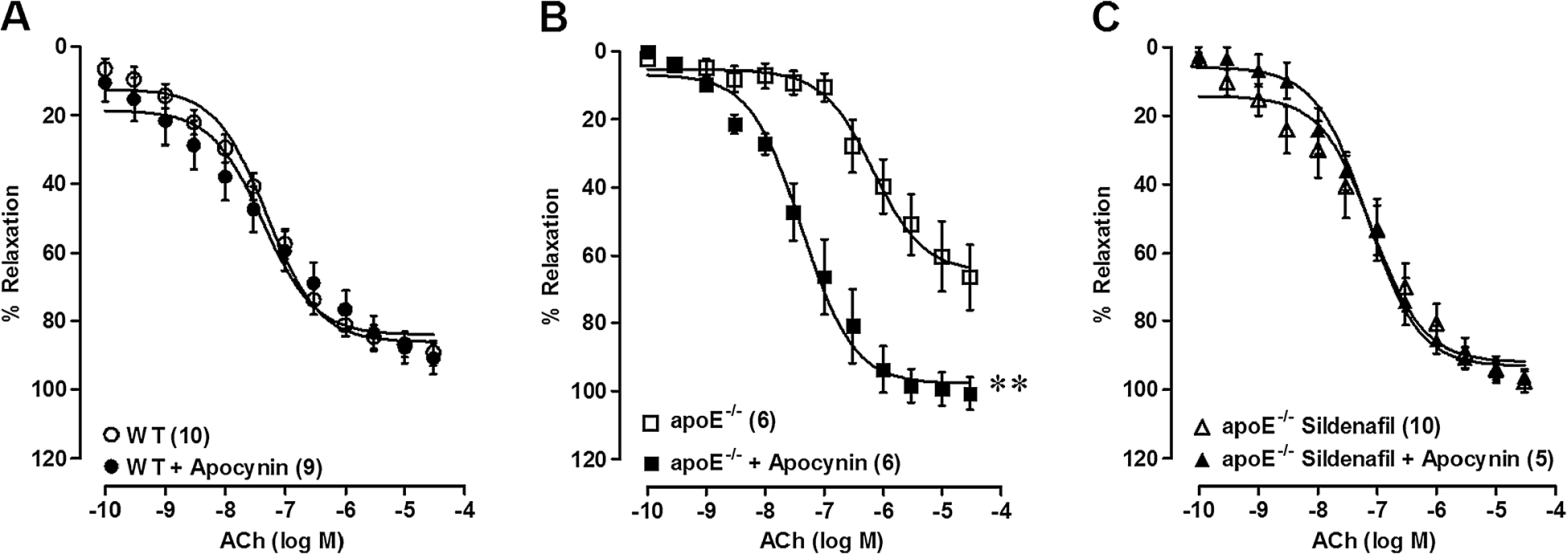
**Participation of reactive oxygen species.** Data show the effect of the blockade of NADPH oxidase activity with apocynin on the cumulative concentration–response curve to acetylcholine (ACh) in untreated and treated apoE^−/−^ mice and wild-type (WT) control mice. Values are mean ± SEM. **p < 0.01 vs. apoE^−/−^ without blockage (one-way ANOVA).

The participation of ROS in the relaxation response to ACh was assessed by incubation of the aortic rings with apocynin. We did not observe significant changes in the vascular reactivity after apocynin blockage in the WT animals, indicating that ROS may not participate in these responses (Table 2, Figure 3A). However, apocynin significantly increased maximal relaxing response and sensitivity to ACh in aortas of untreated apoE^−/−^ mice (Table 2, Figure 3B). In apoE^−/−^ mice treated with sildenafil, the ACh-induced vasorelaxation in the presence of apocynin was similar to that observed in WT mice (Table 2, Figure 3C).

**Figure 4:**
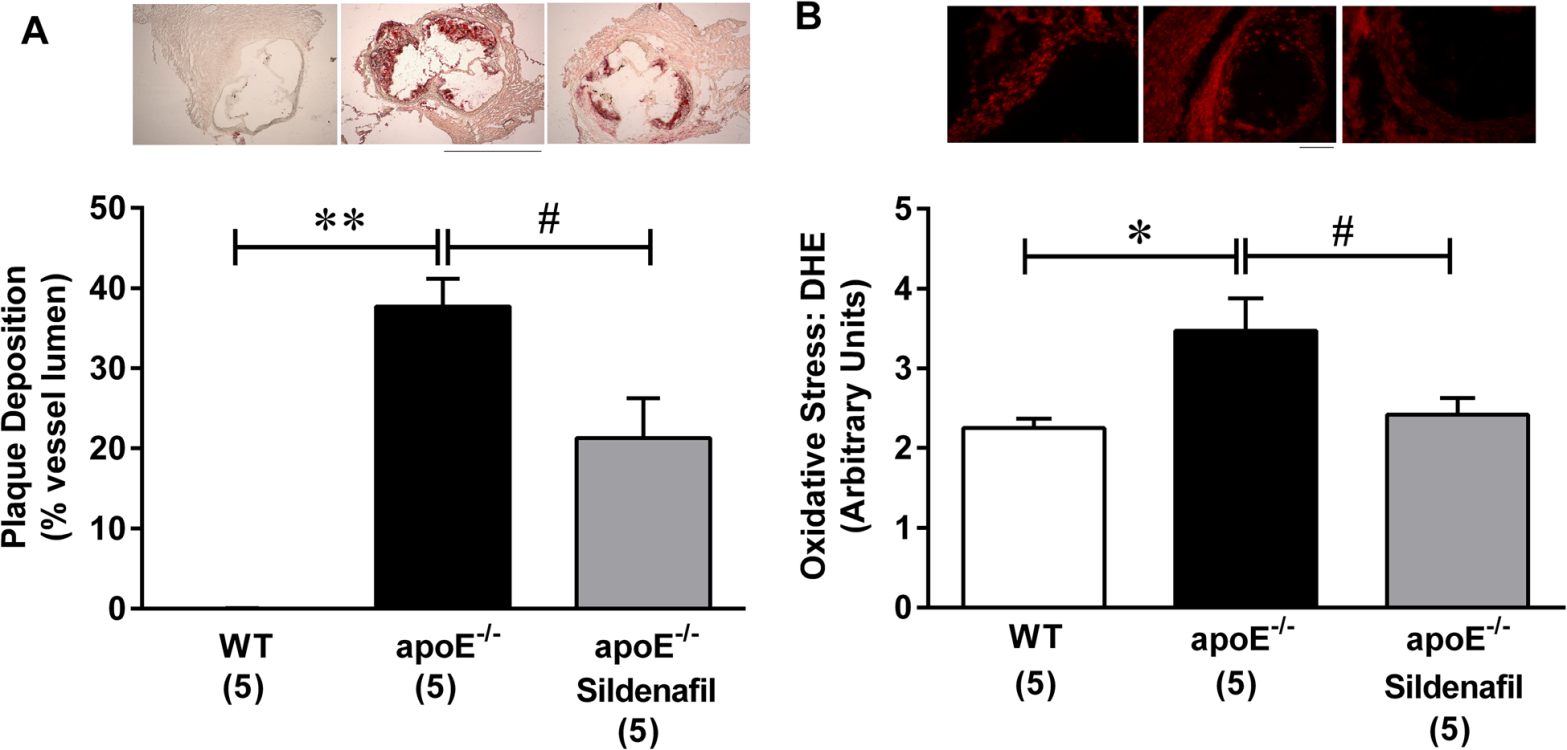
**Effect of sildenafil treatment on plaque deposition and oxidative stress. **(**A**) Bar graph showing mean ± SEM values (in %) of plaque deposition in untreated and treated apoE^−/−^ mice compared to wild-type (WT) control mice. Photomicrographs are typical aorta cross-sections of each group of animals. Scale bar 500 μm. (**B**) Bar graph showing mean ± SEM values of dihydroethidium (DHE) labeling (in arbitrary units) comparing the 3 groups of animals. Photomicrographs above the graph are typical aorta cross-sections showing fluorescent DHE labeling. Scale bar 100 μm. *p < 0.05 and **p < 0.01 untreated apoE^−/−^ vs. WT; ^#^p < 0.05 treated vs. untreated apoE^−/−^ vehicle (one-way ANOVA).

Figure 4 summarizes the results of histological analyses of aorta from the three groups of animals. ApoE^−/−^ mice showed a significant plaque deposition in the aortic root when compared to WT animals (37.7 ± 3.4% vs. 0.0 ± 0.0%, p < 0.01). Sildenafil treatment in the apoE^−/−^ animals caused approximately a 40% reduction of the atherosclerotic plaque deposition (21.3 ± 5.0, p < 0.05; Figure 4A). DHE staining revealed an increased oxidative stress in the aortas from untreated atherosclerotic apoE^−/−^ animals (3.5 ± 0.4, p < 0.05) compared to the WT group (2.30 ± 0.1), and treatment with sildenafil reduced the oxidative stress in aortas from apoE^−/−^ mice (2.4 ± 0.2, p < 0.05 vs. untreated apoE^−/−^ mice; Figure 4B).

## Discussion

This work showed for the first time that chronic inhibition of PDE5 with sildenafil restored endothelial function in the aorta of apoE^−/−^ mice. In addition, sildenafil treatment reduced the atherosclerotic plaque deposition in this large vessel without affecting the plasma cholesterol levels. The amelioration of endothelial function and delayed progression of atherosclerosis that was observed, were mainly regulated through improving the basal NO pathway activation and decreasing ROS production.

As expected, the apoE^−/−^ mice showed higher plasma cholesterol than the WT animals [17-20], exhibiting approximately a 14-fold increase in total cholesterol, as recently reviewed [4]. Interestingly, sildenafil did not change the plasma lipid profile in the treated animals, which was consistent with similar results found elsewhere [21], although it improved the endothelial function. In recent years, there has been a significant improvement in the development of new therapeutics that target cardiovascular risk factors other than hypercholesterolemia [22]. Similar to the results in our present study, a class of statins has been shown to have pleiotropic effects with atheroprotective properties that were independent of cholesterol and were mediated by inhibition of isoprenoids [23]. Hence, we propose that the beneficial vascular effects of sildenafil may offer a new perspective for the use of PDE5 inhibitors in ameliorating endothelial dysfunction and reducing atherosclerosis.

It has been postulated that the major cause of endothelial dysfunction observed in apoE^−/−^ mice is the reduction in the NO pathway, including the increased inactivation of this species by ROS [4,24]. NO is a multifunctional molecule, exerting several potentially anti-atherosclerotic effects, such as antioxidant, anti-inflammatory, anti-platelet and vasodilator [4,25]. Because the atherosclerotic disease is initiated by an endothelial dysfunction, novel therapeutics that potentiate or mimic the increase in NO bioavailability should also be considered. Sildenafil appears to be a promising alternative as it inhibits the degradation of cGMP, the second messenger of NO [4,6,26]. The potential advantage of a therapeutic approach based on inhibition of PDE5 is that, unlike strategies that attempt to increase NO levels (e.g., substrates, co-factors, antioxidants) [27], this one avoids risks associated with excessive NO. At high concentrations, NO is a cytotoxic free radical that increases oxidative stress independent of activation of guanylyl cyclase, thereby contributing to the development of atherosclerosis through overproduction of peroxynitrite (ONOO^-^) [28,29].

In the present study, we observed marked endothelial dysfunction in the aorta of apoE^−/−^ mice, as shown before by others in conductance and resistance vessels [24,25,30,31]. We found that this endothelial dysfunction is related to reduced participation of NO in the vasodilator response to ACh, as demonstrated by a reduced area under the ACh concentration-response curve when blocked with L-NAME; this was in accordance with previous results [25]. It has already been shown that apoE^−/−^ mice exhibit reduced activity of endothelial NO synthase (eNOS), even if the expression of the enzyme is normal [24,32]. Furthermore, some studies have evaluated the hypothesis that the vascular dysfunction in apoE^−/−^ mice may be dependent not only on the reduced bioavailability of NO but also on the altered responsiveness of vascular smooth muscle cells (VSMCs) to NO. We did not observe differences in sodium nitroprusside responses between WT and untreated apoE^−/−^ mice (data not shown), demonstrating that the prejudice of vascular function is not due to a reduction in the sensitivity of smooth muscle cells to NO; this contradicts what was previously reported in the mesenteric arteriolar bed [33] and in the aorta [34-36].

The efficacy of sildenafil in promoting the vasodilator response to ACh was demonstrated in diabetic rats [37] and in a secondhand smoke mouse model [38]; however, this efficacy has not yet been demonstrated in atherosclerotic experimental animals. The novel finding of our study is that sildenafil treatment restored endothelial function in this murine model of atherosclerosis. Once sildenafil main mechanism of action is inhibition of PDE5 in VSMCs, the drug mimics the increase in NO availability. In addition, recent data have shown that sildenafil can increase the expression [39-41] and activity [28,38] of eNOS, which could explain the amelioration of endothelial function and the reduction in atherosclerotic lesions.

Our data provide evidence that NADPH-derived ROS production contributes to oxidative stress in apoE^−/−^ animals, because apocynin ameliorated ACh-induced relaxations in this group as shown previously [42]. The novelty of the present study is that sildenafil treatment restores endothelial function in apoE^−/−^ mice by a reduction in oxidative stress. It has been shown that increased cGMP can inhibit NADPH oxidase expression/activity, thereby reducing ^·^O_2_^-^ production, and consequently restoring NO bioavailability. This antioxidative effect of sildenafil may constitute an additional mechanism for the augmentation of NO-mediated relaxation [26]. These results on the oxidative stress, as assessed by a vascular reactivity study, are in accordance with the *in situ* observations using frozen sections labeled with DHE. In accordance with others [24,43,44], we observed that apoE^−/−^ mice show increased DHE staining, which represents increased ^·^O_2_^-^ production. Our finding is in agreement with another study, which demonstrated a reduction in nitrotyrosine immunostaining (a marker of local oxidative stress, produced by the interaction of ONOO^-^ with tyrosine residues in peptides) in ischemic muscles of apoE^−/−^ animals treated with sildenafil [21].

It has been postulated that atherogenesis is initiated with an endothelial dysfunction, in response to an injury to the endothelium caused by risk factors such as dyslipidemia [1,4]. Thus, interventions that restore endothelial function can potentially reduce atherosclerotic plaque deposition. We observed that sildenafil treatment, in addition to amelioration of endothelial function in hypercholesterolemic apoE^−/−^ mice, is capable of reducing atherosclerosis in the aorta of treated animals by approximately 40%. Atherosclerosis is considered an inflammatory disease, in which the migration of leucocytes through the endothelial wall is a crucial event. Circulating monocytes, attracted by chemoattractants that are released by activated endothelial cells, adhere to the vessel wall by interacting with E-selectin, vascular cell adhesion molecule (VCAM-1) and intercellular adhesion molecule (ICAM-1). This initiates a process of phagocytosis of modified LDL and foam cell formation [45]. Sildenafil can reduce atherosclerosis by interfering with this inflammatory cascade [11] by preventing oxidation of LDL, in addition to improving the endothelial function. A recent report has shown that a dose of sildenafil similar to that used in our study increases the number of bone marrow-derived endothelial progenitor cells in hypercholesterolemic apoE^−/−^ mice [21]. These cells may be involved in the reduction of oxidative stress and apoptosis through cell therapy, as observed by our group recently [43],44], and this finding may offer new insight into similar effective therapies.

In conclusion, we are the first to show that chronic sildenafil treatment restores endothelial function and reduces oxidative stress in a murine atherosclerotic model based on a reduction in plaque deposition in these mice, independent of changes in the lipid profile. This study confirms that sildenafil is a promising novel pharmacologic strategy to treat atherosclerosis, although further studies are necessary to confirm the safety of this pharmacological approach in atherosclerotic patients.

## Competing interests

The authors declare that they have no competing interests.

## Authors’ contributions

CMB and MAL carried out the animal experiments, the data analysis and the statistics and drafted the manuscript. IBSG and ALG contributed to the animal experiments and the data analysis. TMCP and SSM participated designing the study and drafted the manuscript. ECV contributed to the conception, design and supervision of the study, as well as the interpretation of the data. All of the authors have read and approved the final version of the manuscript.
